# Inhibition of STING-mediated type I IFN signaling by African swine fever virus DP71L

**DOI:** 10.1186/s13567-025-01474-3

**Published:** 2025-02-04

**Authors:** Lakmal Ranathunga, Sachini Abesinghe, Ji-Won Cha, Niranjan Dodantenna, Kiramage Chathuranga, Asela Weerawardhana, D. K. Haluwana, Nuwan Gamage, Jong-Soo Lee

**Affiliations:** 1 College of Veterinary Medicine, Chungnam National University, Daejeon, Republic of Korea; 2 Department of Animal Science, Faculty of Agriculture, University of Peradeniya, Peradeniya, Sri Lanka

**Keywords:** African swine fever virus (ASFV), DP71L, SITNG, type I interferon (IFN-I)

## Abstract

African swine fever virus (ASFV) is nucleocytoplasmic large DNA arbovirus and encodes many proteins involved in the interaction with host molecules to evade antiviral immune responses. Especially, evasion strategies of type I interferon (IFN-I)-mediated immune responses are crucial for early ASFV replication. However, there is still a lack of information regarding the immune evasion mechanism of ASFV proteins. Here, we demonstrated that ASFV DP71L suppresses STING-mediated antiviral responses. The conserved phosphatase 1 (PP1) motif of DP71L specifically interact with the C-terminal tail (CTT) of STING and in particular, amino acids P371, L374, and R375 of STING were important for interaction with DP71L. Consequently, this interaction disrupted the binding between STING and TANK-binding kinase 1 (TBK1), thereby inhibiting downstream signaling including phosphorylation of TBK1, STING and IRF3 for antiviral signaling. DP71L significantly interfered with viral DNA induced interferon production and IFN-mediated downstream signaling in vitro. Consistently, knockdown of DP71L enhanced antiviral gene expression in ASFV-infected cells. Taken together, these results highlight the important role of DP71L with respect to inhibition of interferon responses and provide guidance for a better understanding of ASFV pathogenesis and the development of live attenuated ASFV vaccines.

**Supplementary Information:**

The online version contains supplementary material available at 10.1186/s13567-025-01474-3.

## Introduction

During a DNA virus infection, viral DNA is released into the cytosol prior to synthesis of viral proteins. Upon sensing cytosolic viral DNA, cyclic GMP-AMP synthase (cGAS) undergoes a rearrangement within its catalytic pocket, leading to production of the noncanonical intracellular second messenger, 2',3'-cyclic GMP-AMP (i.e., 2',3'-cGAMP or cGAMP), which induces conformational changes upon binding with the endoplasmic reticulum (ER)-resident adaptor protein known as the stimulator of interferon genes (STING). STING, also referred to as transmembrane protein 173 or MPYS/MITA/ERIS, plays a critical role in DNA virus-mediated antiviral immunity [1–3]. When bound to cGAMP, STING translocates from the ER membrane to the Golgi apparatus through the ER-Golgi intermediate compartment, thereby triggering autophagy and activating antiviral immune responses mediated by IFN-I [4]. IFN-I modulates various immune functions, including natural killer and T/B cell activity, and dendritic cell activation, maturation, migration, and survival. It coordinates innate and adaptive immune effector functions, thereby serving as the primary defense mechanism against invading viruses [5]. Therefore, many DNA viruses have evolved inhibitors of STING-mediated IFN-I signaling to facilitate their replication [6–8].

ASFV, which belongs to the genus *Asfivirus* (family, *Asfarviridae*), is a large and complex cytoplasmic double-stranded DNA arbovirus. The genome size of ASFV varies between isolates, ranging from 170 to 193 kbp, and encodes 150 to 167 proteins involved in formation of virus structure, virus replication, and immune evasion. ASFV replicates primarily in the cytoplasm of monocyte-macrophage-lineage cells, specifically in the perinuclear cytoplasmic region known as the “viral factory”, and causes hemorrhagic ASF in domestic pigs [9, 10]. Given the near 100% mortality rate, an outbreak of ASF can result in significant economic losses, and impact global pork supplies and food security. To date, there are no commercial effective vaccines or drugs to combat ASFV [11–13].

Recent studies show that the ASFV is sensitive to IFN [14, 15], and that some ASFV strains, such as Armenia/07, 22653/14, L60, and ASFV/NHV, suppress IFNs and IFN-stimulated genes (ISGs) in infected cells [16–18]. ASFV has developed various strategies to evade the host IFN responses during the replication process, and several ASFV-encoded proteins have been identified as IFN-I antagonists [15, 19–28]. In particular, several ASFV proteins targeting STING have been identified. ASFV L83L recruits Tollip to promote STING degradation through autophagy-lysosomal pathways [29], while D117L binding to STING interferes with TBK1 and nuclear factor Kappa-B kinase ε (IKKε) recruitment [30]. MGF505-7R upregulates autophagy-related Unc-51-like kinase 1 to degrade STING, and MGF505-11R promotes STING degradation through the lysosomal, ubiquitin–proteasome, and autophagy pathways [21, 31]. Recently, we reported a mechanism by which ASFV B175L inhibits STING-mediated antiviral signaling to allow efficient infection [32].

DP71L, the ASFV MyD116 homolog, encodes a 7.7 kDa protein that recruits protein phosphatase 1 (PP1) to dephosphorylate eukaryotic initiation factor-2α (eIF2α), thereby restoring host protein translation and inhibiting apoptosis [33]. A recent report showed that deleting the DP71L gene from virulent ASFV strains attenuates their virulence significantly, thus highlighting its role as a critical factor in vaccine development [34]. While deletion of DP71L reduces ASFV virulence demonstrably, the precise molecular mechanisms by which it contributes to pathogenesis remain to be elucidated. Here, we describe a novel mechanism by which ASFV DP71L suppresses host IFN-I signaling by targeting STING directly, a finding that highlights the crucial role of DP71L in ASFV virulence.

## Materials and methods

### Cell culture

The following cell lines were used: porcine kidney epithelial cells (PK-15) (ATCC CCL-33), HeLa cells (ATCC CCL-2), HEK293T cells (ATCC CRL-11268), Vero cells (ATCC CCL-81), 293-Dual hSTING-A162 cells (InvivoGen, San Diego, CA, USA, 293d-a162), A549 cells (ATCC CCL-185), monkey kidney epithelial cells (MA104) (CRL-2378.1), 3D4/21 cells (ATCC CRL-2843), STING wild-type (STING WT) and STING-knockout (STING KO) HeLa cells. PK-15, HeLa, HEK293T, Vero, A549, MA104, and 293-Dual hSTING-A162 cells were cultured in Dulbecco’s Modified Eagle Medium (DMEM) (Cytiva) supplemented with 10% fetal bovine serum (FBS) (Gibco) and 1% antibiotic/antimycotic (AA) (Gibco). For 293-Dual hSTING-A162 cell culturing selective antibiotics were added according to the manufacturer’s instructions. For the culture of STING KO HeLa cells, Minimum Essential Medium (MEM) (WEL GENE-LM 007–60) was utilized supplement with 10% FBS and 1% AA. Furthermore, 3D4/21 and porcine-immortalized bone marrow-derived macrophages (PIB) cells were cultured in RPMI-1640 medium (Cytiva) supplemented with 10% FBS and 1% AA. For the ASFV infection experiments, primary porcine alveolar macrophages (primary PAM) (Optipharm Inc., Cheongju, Republic of Korea) cells were used and cultured in 10% FBS and 1% Penicillin–Streptomycin (Gibco) added RPMI-1640 medium.

To establish stable cell lines expressing pIRES-Flag (control) or pIRES-DP71L-Flag (DP71L-Flag), 3D4/21, MA104, and PIB cells were transfected with pIRES-Flag (control) or pIRES-DP71L-Flag plasmids with Lipofectamine 2000 (Invitrogen), and positive colonies were selected in 10% FBS containing cell culture media supplementation with 2.0, 4.0, and 0.3 μg/mL of puromycin, respectively.

### Immortalized porcine bone marrow-derived macrophages generation

In this study, we utilized previously established immortalized porcine bone marrow-derived macrophages (pBMDM) [28]. In brief, for immortalization, differentiated pBMDM cells were split into a 24-well cell culture plates at a concentration of 1 × 10^6^ cells/mL in a media containing RPMI-1640 supplemented with 10% FBS, GlutaMAX-I and 1% AA. At the 50–70% cell confluence, cells were infected with lentivirus expressing SV40 large T antigen under the CMV promoter with neomycin marker (amsbio) in the presence of polybrene. The cells were then incubated for 48–72 h in a 5% CO_2_, 37 °C temperature incubator.

After three days, cell media was replaced with complete media containing 800 μg/mL G418 to select positive cells. Immortalization was verified by the PCR amplifying of SV40LT. For that, genomic DNA was isolated using an RNA/DNA mini kit to serve as the template for the PCR. PCR primers for the SV40LT: forward primer—5’-GATGGCTGGAGTTGCTTGGCTACAC-3’ and reverse primer—5’-GCCTGAAATGAGCCTTGGGACTGTG-3’. PCR was performed with an initial denaturation step of 5 min at 95 °C, followed by 35 cycles, each consisting of 30 s at 94 °C, 30 s at 63 °C, and 45 s at 72 °C. Finally, an extension step was maintained at 72 °C for 5 min.

### Quantitative real-time PCR (qRT-PCR)

DP71L-transfected PK-15, or stable 3D4/21, and relevant control cells were cultured in 12-well tissue culture plates at a density of 3 × 10^5^ cells per well. The plates were then incubated in a humidified atmosphere containing 5% CO_2_ at a temperature of 37 °C. After 12 h of incubation, the cells were infected with ADV-GFP (MOI = 1.0). Following infection, cells were harvested at two-time points, specifically 12 and 24 h post-infection (hpi), and stored at −80 °C until further analysis. To extract the total RNA, we employed the Machery Nagel Nucleospin RNA kit (790,955). Subsequently, complementary DNA (cDNA) was synthesized from the isolated RNA using Compact cDNA Synthesis Kit (SMARTGENE; SG-cDNAC100). Finally, real-time polymerase chain reaction was conducted to measure and quantify the levels of different cDNA samples. This was achieved using the Sybr Green Q-PCR Master Mix (SJ Bioscience; SG-SYBR-500) according to the manufacturer’s instructions. The qRT-PCR was performed on a Rotor-Gene Q instrument (Qiagen). The mRNA expression levels were analyzed according to the delta–delta CT (2^−ΔΔCT^) method, and β-actin or glyceraldehyde-3-phosphate dehydrogenase (GAPDH) was used as an internal housekeeping gene for normalization. For qRT-PCR, we used specific primer sets designed for each gene of interest, the details of which can be found in Table 1.

**Table 1:** **List of primers used for real-time PCR**

Target gene	Forward primer	Reverse primer
DP71L	ATGGGGAGGCGGCGCAAAAA	TTACTGCTGCTCCAGTAGCT
CP204L	TCTTTTGTGCAAGCATATACAGCTT	TGCACATCCTCCTTTGAAACAT
I73R	ATGGAGACTCAGAAGTTGA	GTTTTTCCGTATCCAAAGC
B646L	CCCAGGGGATAAAATGACTG	CACTGGTTCCCTCCACCGATA
IFN-β	AAATCGCTCTCTGATGTGT	TGCTCCTTTGTTGGTATCG
IFN-γ	CCATTCAAAGGAGCATGGAT	ATCCATGCTCCTTTGAATGG
IL-6	CACCGGTCTTGTGGAGTTTC	GTGGTGGCTTTGTCTGGATT
IL-1β	GGGACTTGAAGAGAGAAGTGG	CTTTCCCTTGATCCCTAAGGT
TNFα	TCACAGGGCAATGATCCC	GGGATCATTGCCCTGTGA
MCP-1	CAGAAGAGTCACCAGCAGCA	TCCAGGTGGCTTATGGAGTC
MX-1	TAGGCAATCAGCCATACG	GTTGATGGTCTCCTGCTTAC
ISG15	AAATCGCTCTCCTGATGTGT	TGCTCCTTTGTTGGTATCG
ISG54	ATGTGCACAGCAATCATGAGT	TTCCTCAGCTAAAGATACTAG
ISG56	CTGACTCACAGCAACCATG	CTTTCAGGTGTTTCACATAGG
β-Actin	CTCGATCATGAAGTGCGACG	GTGATCTCCTTCTGCATCCTGT

### Plasmids, chemicals, and transfection

To obtain the STING sequence, template DNA was subjected to PCR amplification. The resulting amplicons were then cloned into pIRES-Flag (STING-Flag), pEXPR-Strep (STING-Strep), and pEBG (STING-GST) vectors. Similarly, the complete sequence of the ASFV DP71L gene (NC_044959.2: from 185,028 to 185,240) was cloned into pIRES-Flag (DP71L-Flag), pEXPR-Strep (DP71L-Strep), and pEBG vectors (DP71L-GST). For further analysis, domain constructs of STING (STING amino acid (aa) 1–145, aa 1–185, and aa 1–340) and DP71L (DP71L aa 1–7 and aa 1–54) were cloned into the pEBG vector. To study the impact of specific mutations on STING, single-site mutants (S366A, P371Q, L374A, and R375A) were generated. The Mutation Generation System Kit (Thermo Scientific; F701) was employed, following the manufacturer's instructions. The resulting mutants were subsequently cloned into the pEXPR-Strep vector. The PCR primers used for site-directed mutagenesis can be found in Table 2. A range of commercially available reagents and supplies were procured to support our experimental procedures. cGAMP (InvivoGen), GlutaMAX Supplement (Gibco), Trypsin–EDTA (Gibco), Normocin-Antimicrobial Reagent (InvivoGen), Puromycin (InvivoGen), Lipofectamine 2000 (Invitrogen), Polyethyleneimine/PEI (Polysciences; 9002-98-6/26913-06-4), Protein A/G PLUS-Agarose (Santa Cruz Biotechnology; sc-2003), Halt Protease Inhibitor Cocktail (Thermo Scientific; 78429), Sepharose 6B (GE Healthcare; 17011001), Glutathione-conjugated Sepharose 4B beads (Cytiva), Strep-Tactin Sepharose resin (IBA Lifesciences; 2–1201-002), and Quanti-Luc (Invivogen). For plasmid transfection, we used PEI for HEK293T cells and 293-Dual hSTING-A162, and Lipofectamine 2000 for all other cell lines, strictly adhering to the instructions provided by the respective manufacturers. Lipofectamine RNAiMAX (Invitrogen) was chosen for cGAMP and siRNA transfection.

**Table 2:** **List of primers used to generate STING mutations**

Target gene	Forward primer	Reverse primer
STING S366A	CCTGAGCTCCTCATCGCTGGAATGGAAAAGCCC	GGGCTTTTCCATTCCAGCGATGAGGAGCTCAGG
STING P371Q	CAGTGGAATGGAAAAGCAACTCCCTCTCCGCACG	CGTGCGGAGAGGGAGTTGCTTTTCCATTCCACTG
STING L374A	AAAGCCCCTCCCTCTCCGCACGGATTTCTCTTGA	TCAAGAGAAATCCGTGCGGAGAGGGAGGGGCTTT
STING R375A	CTCCCTCTCGCCACGGATTTCTCT	AGAGAAATCCGTGGCGAGAGGGAG

### Virus infection and replication assay

Viruses used in this study were Adenovirus (ADV-GFP), Herpes Simplex Virus (HSV-GFP), and Vaccinia Virus (VACV-GFP), and these viruses were propagated in PK-15 cells (ADV-GFP) and Vero cells (HSV-GFP and VACV-GFP). Each virus titers were determined using plaque assays. Virus infection experiments were conducted in 12-well cell culture plates and the virus infection into cells at a specific MOI was done in reduced serum (1% FBS)-containing medium for 2 h and incubated in 5% CO_2_, 37 °C temperature incubator. After the infection period, the supernatants, which contain the non-infected viruses were replaced with fresh culture medium containing 10% FBS. GFP fluorescence was visualized at 24 hpi using an Olympus inverted fluorescence microscope (Olympus IX71, Tokyo, Japan) with a magnification of × 200. To quantify the fluorescence intensity, cells were collected with the supernatants at 12 hpi and 24 hpi and centrifuged at 3000 rpm for 3 min. Cell supernatants of each sample were used for ELISA to check cytokine secretions. Cell pellets were suspended in 300 µL of phosphate-buffered saline (PBS), and fluorescence of each sample were analyzed using a fluorescence modulator (GloMax-Multi Detection System, Promega).

Additionally, standard plaque assays were conducted using A549 cells (for ADV-GFP and VACV-GFP) and Vero cells (for HSV-GFP) to determine the viral titers. Initially, virus infected cell supernatants were collected from relevant treatment samples at 12 hpi and/or 24 hpi and transferred them into A549 or Vero cells grown in 12-well cell culture plates separately. Each initial virus containing supernatant samples were serially diluted (until 10^–10^) in reduced DMEM (1% FBS), and incubated for a period of 2 h in 5% CO_2_ 37 °C temperature incubator. After the incubation, the inoculum was replaced with DMEM containing 0.1% agarose (Sigma-Aldrich) and further incubation was done until 36 h in similar incubation conditions. Finally, plaque formation was examined under the × 200 magnification using Olympus inverted fluorescence microscope. Virus titers were assessed by determining the number of plaque-forming units (PFUs) and incorporating the dilution factor into the calculations. For the ASFV infection experiment to assess the DP71L transcription profiles, primary PAM cells were infected with 0.5 MOI of ASFV (Korea/wild boar/Hwacheon/2020–2287). Subsequently, cell pellets were harvested at 0, 3, 6, 9, 12, 15, and 18 hpi to extract RNA, and synthesize cDNA. Finally, qRT-PCR analysis was performed using DP71L, ASFV I73R (an early gene) and B646L (a late gene) specific primers (Table 1) to assess the gene transcription kinetics. Experiments involving ASFV infection were carried out in accordance with the Standard Operating Procedure (SOP) in the biosafety level 3 (BSL-3) laboratory at the National Institute of Wildlife Disease Control and Prevention (NIWDC) in Korea.

### Small interfering RNA (siRNA) knockdown

ASFV DP71L specifically targeting siRNA was designed and synthesized by Bioneer (Daejeon, Republic of Korea). Primary PAM cells, with a cell number of 1 × 10^6^ cells per well in 24-well plates, were transfected with either control (siControl) or DP71L (siDP71L) siRNA using RNAiMAX. The target sequence for siDP71L is GACGAACGACGCGAAGCAU, and for siControl, it is AUGCUUCGCGUCGUUCGUC. At 6 h post-transfection (hpt), cells were either left uninfected or infected with ASFV at a 0.5 MOI. The qRT-PCR method was used to detect the mRNA expression levels of the target ASFV genes, as well as antiviral gene transcriptions (Table 1). ASFV infected experiments were conducted according to the Standard Operating Procedure (SOP) in BSL-3 laboratory of the NIWDC in Korea.

### Immunoprecipitation, immunoblot analysis and antibodies

For immunoblot (IB) analysis, cells were harvested 36 h after transfection with relevant plasmids and lysed using lysis buffer containing protease inhibitor cocktail, phosphatase inhibitor cocktail (Sigma), and radio-immunoprecipitation assay (RIPA) buffer. The RIPA buffer composition included 50 mM Tris–HCl, 150 mM NaCl, 0.5% sodium deoxycholate, and 1% IGEPAL. The lysates were subjected to sonication (30% amplitude, 10 s, three cycles for 500 μL of lysate) and centrifuged at 12 000 rpm, 4 °C, for 10 min. The resulting whole-cell lysates (WCLs) were mixed in a 1:1 ratio with Sample Buffer, Laemmli 2 × concentrate (Sigma-Aldrich; S3401), heated at 100 °C for 10 min, and then analyzed by SDS-PAGE. For pull-down assays (PD), the WCLs were subjected to pre-clearance by adding Sepharose 6B, followed by incubation in a rotator at 4 °C for 2 h. After centrifugation at 8000 rpm and 4 °C for 5 min, the WCLs were incubated overnight at 4 °C with Strep-Tactin Sepharose resin or Glutathione-conjugated Sepharose 4B beads. Subsequently, the beads were collected by centrifugation (8000 rpm, 4 °C for 4 min) and subjected to washing with NP40 lysis buffers using relevant washing conditions. Beads were further cleared by needle suction for removing remain washing buffers. The resulting beads were mixed with sample buffer (around 60 μL), heated at 100 °C for 10 min and associated proteins were observed by SDS-PAGE. In parallel, for antibody immunoprecipitations (IP), WCLs were incubated with respective primary antibodies and after 12 h, protein A/G PLUS-Agarose beads 25 μL were added into each WCL samples, which were containing primary antibodies. Next, they were further incubated for 4 h at 4 °C rotator. Beads were prepared as mentioned in bead pull-down assays, and associated proteins were observed by SDS-PAGE.

SDS-PAGE proteins were then transferred to a polyvinylidene difluoride (PVDF) membrane by a Trans-Blot semi-dry transfer cell system (Bio-Rad, Seoul, South Korea). Following protein transfer, membranes were blocked with 5% bovine serum albumin (Georgiachem, Norcross, GA, USA, BS1005) for 1 h to prevent non-specific binding, and incubated overnight with the primary antibodies at 4 °C on a rocker. The following day, the protein-transferred PVDF membranes were washed with TBST and then incubated with horseradish peroxidase-conjugated (HRP) secondary antibodies (GeneTex, Irvine, CA, USA, GTX213111-01) for 2 h at room temperature. After another washing with TBST, all the PVDF membranes were visualized using an enhanced chemiluminescence detection system (ECL-GE Healthcare, Little Chalfont, UK) employing a Las-3000 mini Lumino Image Analyzer. The antibodies used in this study were obtained from Cell Signaling Technology and included STING (D2P2F; 13647), TBK1/NAK (D1B4; 3504), STAT1 (42H3; 9175), NF-κB p65 (D14E12; 8242), IRF3 (D83B9; 4302), IκBα (9242), pSTING (85735), pTBK1/NAK (D52C2; 5483), pSTAT1 (58D6; 9167), pNF-κB p65 (93H1; 3033), pIRF3 (4D4G; 4947), pIκBα (14D4; 2859), and Flag (M2) (8146). Additionally, other antibodies used in the study are Alexa Flour 488 (Abcam; 150077), Alexa Flour 647 (Abcam; 150079), StrepMAB-Classic HRP conjugate (IBA Lifesciences; 2–1509-001), β-actin (Santa Cruz Biotechnology; sc-47778), and GST (Santa Cruz Biotechnology; sc-138).

### Semi-denaturing detergent agarose gel electrophoresis (SDD-AGE) assay

The SDD-AGE assay was performed following a previously described protocol [35], with certain modifications. HEK293T cells were cultured in 6-well plates and transfected with STING-Strep and DP71L-Flag plasmids as indicated. The following day, the cells were stimulated with 4 μg/well of cGAMP ligand for 4 h. Subsequently, the cells were washed with PBS, and lysed using RIPA buffer supplemented with phosphatase and protease inhibitor cocktail. WCLs were subjected to SDS-PAGE, and glycine-eluted immunoprecipitated proteins were resolved using 1.5% SDD-AGE.

### Confocal imaging

HeLa or PK-15 cells were cultured in an 8-well chamber slide (ibidi; 80,826) and fixed with 4% paraformaldehyde for 20 min at room temperature. Following PBS washing, the cells were treated with 100% methanol at −20 °C for 20 min to allow permeabilization. Subsequently, a blocking solution of 2% BSA in PBS was applied for 1 h at room temperature to prevent non-specific binding. The cells were then exposed to the appropriate primary antibodies and incubated overnight at 4 °C. Next day, the cells were washed three times with PBST and exposed to the corresponding secondary antibody. After three additional PBST washes, the cells were stained with DAPI (4’,6-diamidino-2-phenylindole) (Invitrogen) for 10 min at room temperature. Images were captured using a Leica Dmi8 microscope and analyzed using LAS-X software (version 3.7.1.21655).

### Luciferase assays

HEK293T cells were seeded into 12-well tissue culture plates and transfected with the IFN-β luciferase (firefly) plasmid, along with the TK-Renilla luciferase (Renilla) reporter plasmid and corresponding molecules (STING, TBK1, IKKε or IRF3) using PEI. Furthermore, DP71L pIRES plasmid or control vector was transfected in dose-dependent manner. After 24 h from the transfection, supernatants were removed and cell layers were rinsed with PBS and lysed with 1 × passive lysis buffer (Promega; E194A) for 15 min. Subsequently, the luciferase activity was measured using the Dual-Luciferase Reporter Assay System (Promega; E1980) following the manufacturer's instructions. The reported values represent the firefly luciferase activity normalized to the Renilla luciferase activity. Furthermore, the 293-Dual hSTING-A162 cells were used to analyze the IFN-β luciferase activity induced by poly(dA:dT), cGAS and cGAMP. Poly(dA:dT) (InvivoGen) and cGAMP ligands were treated with Lipofectamine 2000 and Lipofectamine RNAiMAX, respectively. Here, QUANTI-Luci were employed to assess the luciferase activity according to the manufacturer’s instructions.

### In vitro binding assay

HEK293T cells were transiently transfected with a plasmid encoding Flag-tagged STING. After 36 h, cells were lysed, and STING-Flag was immunoprecipitated and eluted with glycine buffer. Separately, HEK293T cells were transfected with either DP71L-Strep or Strep-tagged control. At 36 h post-transfection, the lysate was incubated with Strep-Tactin Sepharose resin overnight in 4 °C rotator. The resin was then subjected to washing with NP40 lysis buffers, and further cleared by needle suction for removing remain washing buffer. Next resin immorbilized Strep-tagged proteins (Control and DP71L) were incubated with purified STING-Flag protein in a binding buffer (50 mM Tris–HCl, pH 7.5, 300 mM NaCl) at 37 °C for 2 h. Subsequently, the resin was washed and removed the unbound proteins. Finally, the bound proteins were eluted by boiling in SDS-PAGE sample buffer, and samples were subjected to SDS-PAGE and immunoblotting with the appropriate antibodies.

### Enzyme-linked immunosorbent assay (ELISA)

An ELISA was conducted to measure the levels of secreted pro-inflammatory cytokines and IFNs in the culture supernatants. Commercial kits from CUSABIO (CSB-E09890p for porcine IFN-β and CSB-E06786p for IL-6) were utilized for the analysis, following the manufacturer’s instructions.

### Mass-spectrometry

The sample preparation procedure was performed based on a previously optimized method with minor modifications [36]. Briefly, HEK293T cells transfected with DP71L-GST or GST control vector plasmids were collected into lysis buffer 50 mM Tris–HCl, 150 mM NaCl, 0.5% sodium deoxycholate, and 1% IGEPAL supplemented with a protease inhibitor and phosphatase inhibitor cocktail at the 36 hpt. Next, cell pellets were subjected to sonication and removed cell debris by centrifugation. Lysates were pre-cleared by adding sepharose 6B beads and incubated for 2 h at 4 °C. These pre-cleared cell lysates were subjected to a pull-down assay using GST beads overnight at 4 °C. After washing the GST resin with five times in lysis buffer and four times in PBS, the proteins bound to the resin were eluted using western blot sample buffer. For protein separation based on molecular weight, 4–15% NuPAGE gels (Invitrogen; NP0323PK2) were utilized, followed by silver staining. Finally, the protein bands present in the gel were subjected to mass spectrometry analysis.

### Statistical analysis

The data are presented as means and standard deviations (SD) and represent at least two independent experiments. Statistical analysis was performed with the Student’s *t*-test in GraphPad Prism 6 software. Asterisks in figures indicate statistical significance as follows: *, *P* < 0.05, **, *P* < 0.01, ***, *P* < 0.001, or ****, *P* < 0.0001.

## Results

### DP71L negatively regulates antiviral immune responses

**Figure 1:**
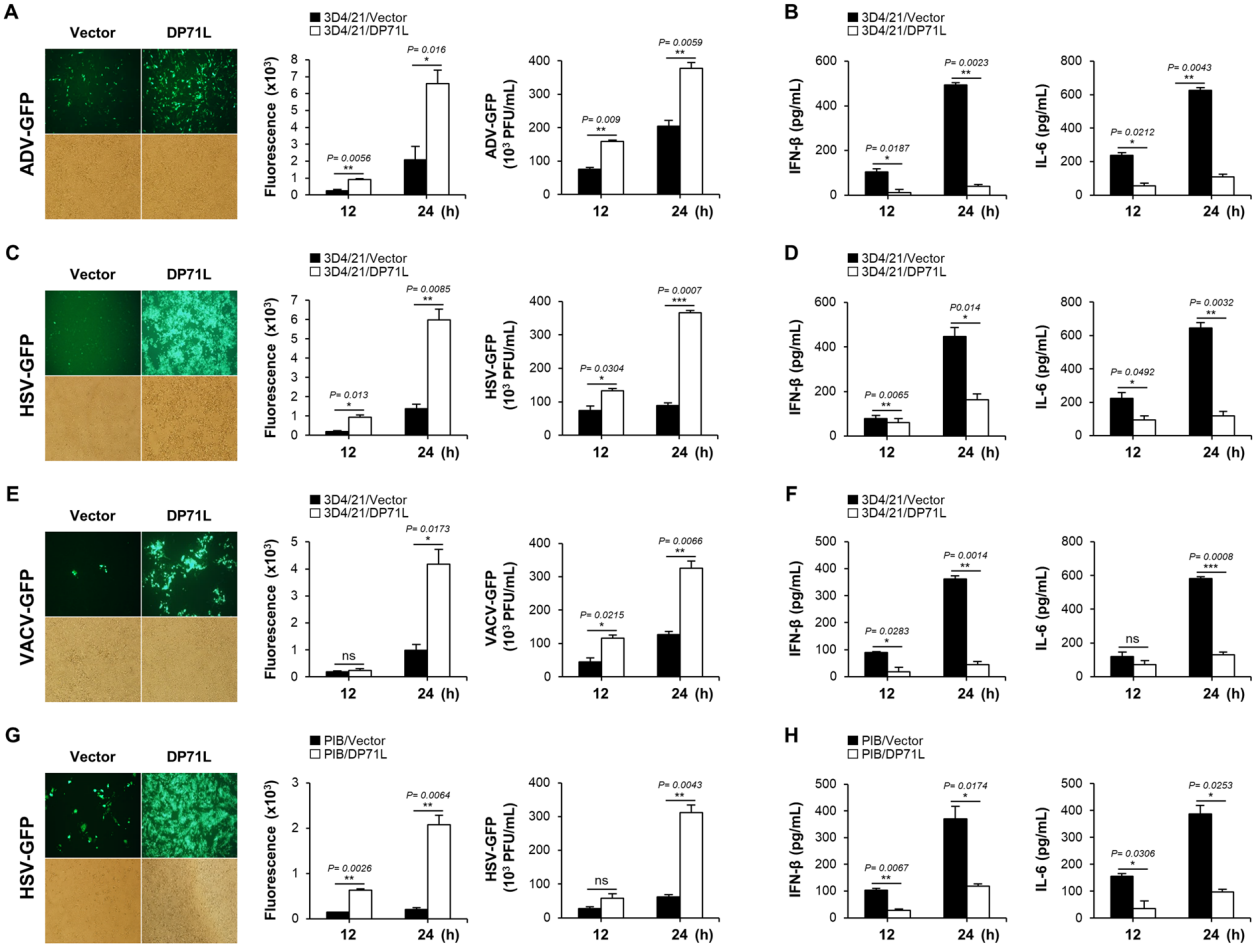
**DP71L downregulates antiviral immune responses in DP71L stably expressing cells. A, C, and E** 3D4/21 cells stably expressing DP71L-Flag or control vector, were infected with ADV-GFP **A**, HSV-GFP **C**, or VACV-GFP **E** (MOI = 1.0). Fluorescence microscopy was used to capture GFP images at 24 hpi, while the fluorescence levels were measured at both 12 and 24 hpi using a fluorescence modulator. The viral titers of each sample were determined through standard plaque assays conducted in A549 and Vero cells. **B**, **D**, and** F** Additionally, the concentrations of porcine IFN-β and IL-6 in the cell culture supernatant collected at 12 and 24 hpi were quantified using ELISA. **G** DP71L-Flag stably expressing PIB cells were infected specifically with HSV-GFP and GFP images were captured at 24 hpi. Fluorescence levels were measured at 12 and 24 hpi and the virus titers were determined by plaque assay, in Vero cells. **H** IFN-β and IL-6 secretion levels were quantified in cell supernatants using ELISA. The data represent at least two independent experiments with similar results, and the values are expressed as means ± SD for two biological replicates. Student’s *t*-test: *, *P* < 0.05; **, *P* < 0.01; ***, *P* < 0.001; ns, not significant.

Viruses have evolved sophisticated strategies, to circumvent IFN signaling and enhance their survival and replication within the host. Similarly ASFV employs range of strategies to inhibit host IFN responses and host cytokine production. To evaluate the potential IFN signaling modulatory ASFV proteins, we used a dual-luciferase reporter assay to screen an ASFV gene library, comprising 60 Flag-tagged ASFV genes, and identified DP71L as one of the seven ASFV genes capable of inhibiting STING-mediated induction of IFN-β luciferase activity [32]. Thereby, initially we investigated the potential effect of DP71L on DNA virus infection by transfecting DP71L expression plasmid into PK-15 cells (Additional file 1A). Subsequently, these cells were infected with GFP-tagged DNA viruses, including ADV-GFP, HSV-GFP, or VACV-GFP to assess the virus replication [32]. Notably, DP71L-overexpressing PK-15 cells exhibited significantly higher GFP absorbance and viral titers than control cells (Additional files 1B, D and F). Furthermore, we performed an ELISA to analyze secretion of IFN-β and IL-6 in virus-infected cell supernatants, interestingly, DP71L-overexpressing cells demonstrated lower cytokine secretion than control cells (Additional files 1C, E and G). To expand our investigation, we prepared MA104 cells, 3D4/21 cells and PIB cells stably expressing DP71L (Additional files 2A–C). These cells were subsequently infected with the GFP-tagged viruses, resulting in outcomes similar to those in PK-15 cells, i.e., increased virus replication and reduced levels of IFN-β and IL-6 secretion compared with control cells (Figure  1 and Additional files 2D–I). Overall, our findings suggest that ASFV DP71L acts as a negative regulator of IFN-I and pro-inflammatory cytokine production while enhancing viral DNA replication in macrophages and epithelial cell lines.

### DP71L inhibits IFN pathway signaling and transcription of antiviral genes

**Figure 2:**
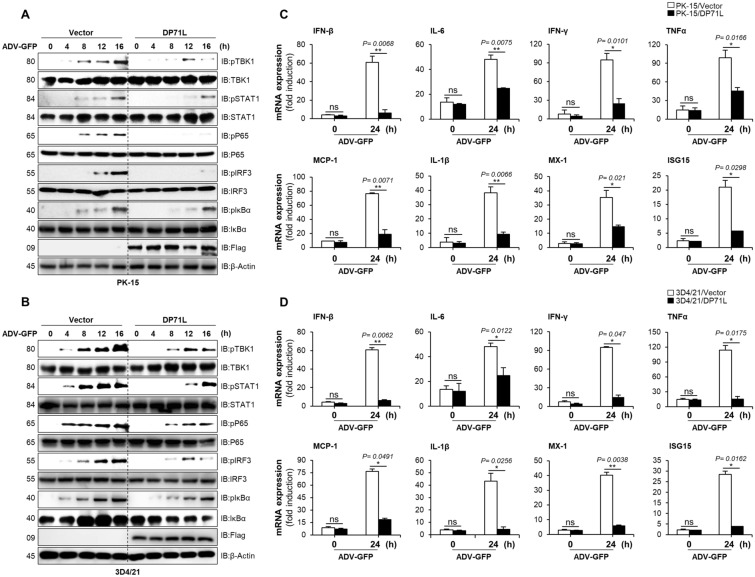
**DP71L inhibits antiviral immune signaling and the transcription of antiviral genes. A and B** DP71L-Flag or control vector transiently and stably expressing PK-15 **A** and 3D4/21 **B** cells were infected with ADV-GFP (MOI = 1.0) and harvested at the indicated time points. Immunoblotting was performed to observe the phosphorylated and intact forms of TBK1, STAT1, p65, IRF3, IκBα, and the expression of DP71L-Flag. The internal control, β-actin, was used to ensure equal protein amounts in the samples. **C and D** DP71L-Flag or control vector transiently and stably expressing PK-15 **C** and 3D4/21 **D** cells were infected with ADV-GFP at a 1 MOI and harvested at indicated time points. The total RNA was extracted from the cells, and qRT-PCR was performed to assess the transcription levels of antiviral genes. Protein sizes are expressed in kilodaltons (kDa). All the data are representative of at least two independent experiments, each with similar results and the values are expressed as means ± SD for two biological replicates. Student’s *t*-test: *, *P* < 0.05; **, *P* < 0.01; ns, not significant.

To determine the effect of DP71L on antiviral signaling cascades, we examined phosphorylation of key molecules involved in the signaling pathway, including TBK1, STAT1, IRF3, p65 (RelA), and NF-κB inhibitor alpha (IκBα), in DP71L-transfected PK-15 cells and DP71L stably expressing 3D4/21 cells following infection with ADV-GFP. Cell samples were collected at 0, 4, 8, 12 and 16 hpi and examined by immunoblotting. As shown in Figures  2A and B, phosphorylation of TBK1, STAT1, IRF3, p65, and IκBα was significantly lower in DP71L-expressing PK-15 and DP71L stably expressing 3D4/21 cells than in their respective control cells at the indicated time points. This observation indicates that DP71L expression suppresses phosphorylation of these molecules during viral DNA infection. Furthermore, we assessed the transcriptional effect of DP71L on IFN-β and ISGs in DP71L-transfected PK-15 and DP71L stably expressing 3D4/21 cells. To do this we infected cells with ADV-GFP, harvested them at indicated time points, and performed qRT-PCR using primers specific for the target genes. The expression levels of mRNA encoding IFN-β and other ISGs were significantly lower in DP71L-transfected PK-15 and DP71L stably expressing 3D4/21 cells than in control cells (Figures 2C and D), suggesting that ASFV DP71L negatively regulates the IFN-I signaling pathway and expression of antiviral genes in response to infection with viral DNA.

### DP71L directly interacts with STING

**Figure 3:**
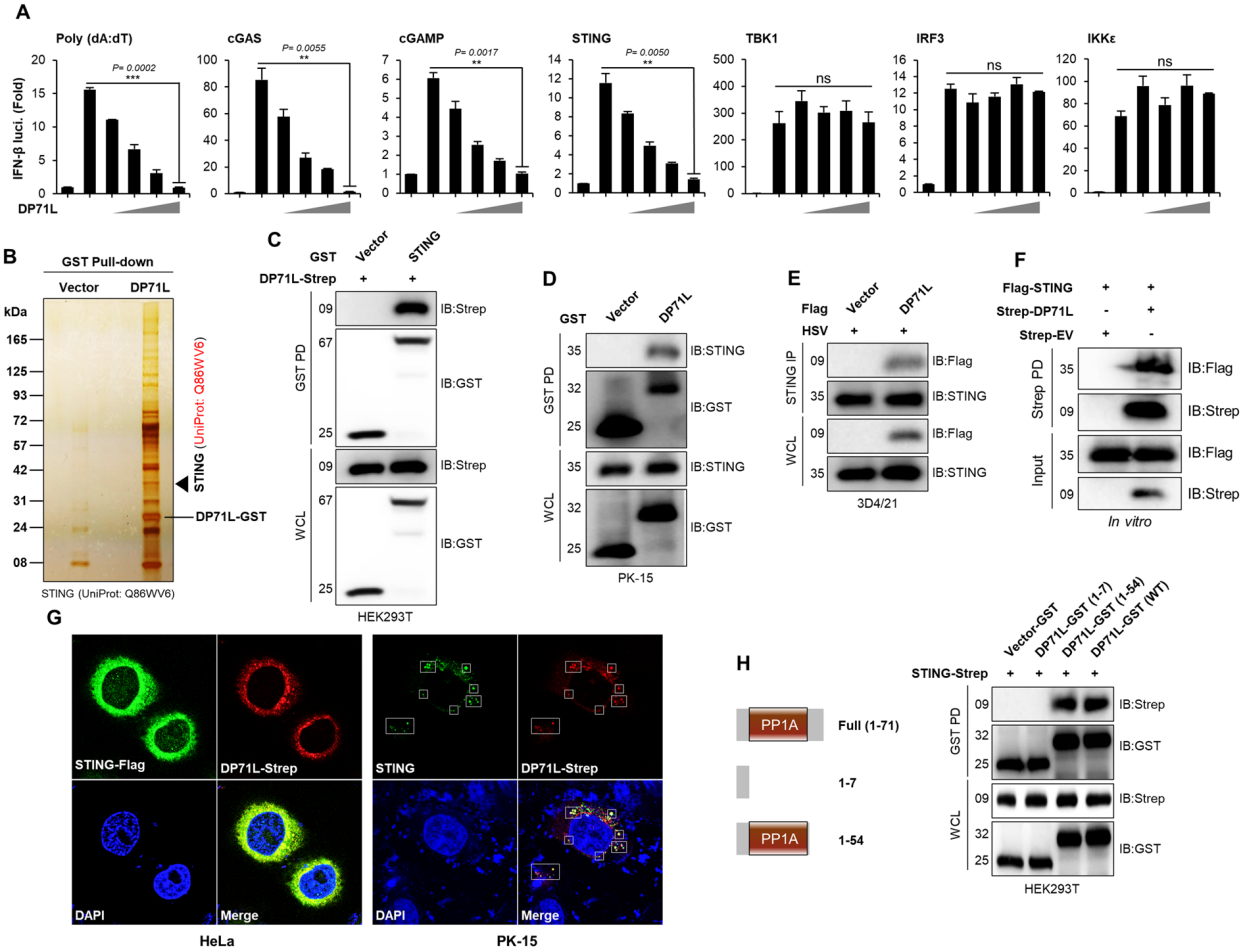
**DP71L interacts with STING. A** HEK293T cells were co-transfected with the IFN-β promoter, TK-renilla, plasmids of STING, TBK1, IRF3, and IKKε, and DP71L-Flag for 24 h. The luciferase activity was measured. Results are demonstrated relative to the Renilla luciferase activity. Additionally, 293-Dual hSTING-A162 cells were transfected with DP71L-Flag plasmid, and transfected with poly(dA:dT), 3xFlag-cGAS plasmid or cGAMP for 12 h. The expression of Lucia luciferase, was determined using the Quanti-Luc assay. **B** The DP71L-GST pull-down assay in HEK293T cells, followed by silver staining and mass spectrometry analysis. **C** HEK293T cells transfected with DP71L-Strep and STING-GST plasmids. Subsequently, cell lysates were subjected to GST PD, and immunoblotted with anti-Strep and anti-GST antibodies. **D** PK-15 cells transfected with DP71L-GST and control plasmids, or **E** 3D4/21 cells stably expressing DP71L-Flag or control plasmids, were stimulated with HSV-GFP (MOI = 1.0). After 24 hpi, cells were harvested and subjected to GST PD or immunoprecipitation with STING antibody, respectively. Immunoblotting was performed with indicated antibodies. **F** In vitro binding assay was conducted by purified STING-Flag protein incubation with Strep-resin immobilized DP71L-Strep or control-Strep proteins. Bound proteins were eluted and immunoblotted with indicated antibodies. **G** Confocal microscopy assays were conducted to investigate the co-localization of DP71L and STING proteins in overexpression and endogenous conditions in HeLa and PK-15 cells. After plasmid transfection, cells were stimulated with VACV wild-type virus. The nuclei were stained with DAPI, and the rectangle indicates the co-localization of DP71L and STING proteins at the endogenous level. **H** HEK293T cells were transfected with GST-tagged DP71L domain constructs and the STING-Strep plasmid. After 36 h, cells were harvested, and WCLs were subjected to a GST PD, followed by immunoblotting with indicated antibodies. Protein sizes are indicated in kDa. The data represent at least three independent experiments with similar results, and the values are expressed as means ± SD for two biological replicates. Student’s *t*-test: **, *P* < 0.01; ***, *P* < 0.001; ns, not significant.

To identify the specific target of the cGAS-STING signaling cascade regulated by DP71L, we performed a series of dual-luciferase reporter assays. In these assays, DP71L was co-expressed with IFN pathway molecules. Our findings revealed that DP71L dose-dependently inhibited IFN-β promoter activity mediated by Poly(dA:dT), cGAS, cGAMP, and STING. However, we found no significant changes in the IFN-β promoter activity mediated by TBK1, IRF3, or inhibitor of IKKε, even in the presence of increasing doses of DP71L (Figure 3A). This suggests that DP71L specifically targets molecules upstream of TBK1. Next, we expressed GST-tagged DP71L in HEK293T cells, and performed a large-scale GST pull-down assay to identify interacting host proteins. Mass spectrometry analysis identified STING (UniProt: Q86WV6) as a candidate target of DP71L (Figure 3B), which led us to hypothesize that DP71L interacts with STING. To confirm this hypothesis, we performed an immunoprecipitation assay in HEK293T cells using Strep-tagged DP71L and GST-tagged STING. As expected, an interaction between DP71L and STING was observed (Figure 3C). Additionally, immunoprecipitation experiments demonstrated the binding of endogenous STING in DP71L-expressing PK-15 and stable 3D4/21 cells (Figure 3D, E). Similar results were obtained by in-vitro binding assay (Figure 3F). Confocal microscopy revealed co-localization of DP71L with overexpressed or endogenous STING in HeLa and PK-15 cells, further supporting an interaction between DP71L and STING (Figure 3G). We successfully identified the specific domain within DP71L that interacts with STING through immunoprecipitation of GST-tagged DP71L domains (aa 1–7 and aa 1–54). The data show that the conserved PP1 domain of DP71L interacts specifically with STING (Figure 3H). Collectively, these results indicate that STING is a direct target of ASFV DP71L, which facilitates suppression of STING-mediated antiviral signaling.

### DP71L inhibits the STING-TBK1 interaction

**Figure 4:**
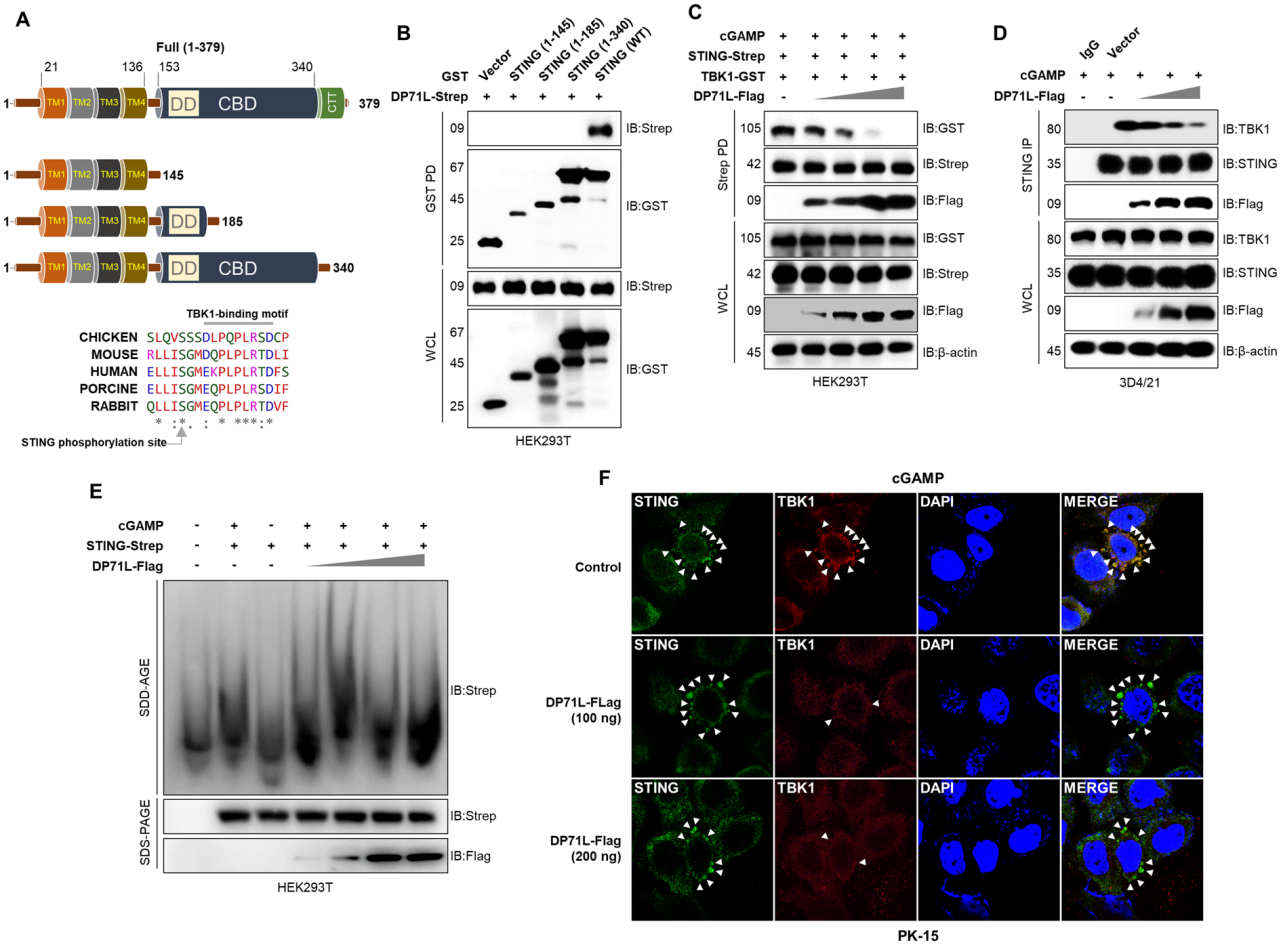
**DP71L inhibits STING-TBK1 interaction. A** Domain organization of STING, highlighting GST-tagged aa 1–145 (TM), aa 1–185 (both TM and DD), aa 1–340 (TM, DD and CBD) and STING-WT. TBK1 binding motif and TBK1 phosphorylation site on STING CTT are conserved among different species. **B** HEK293T cells were transfected with GST-tagged STING domain constructs and the DP71L-Strep plasmid. At 36 hpt, WCLs were subjected to a GST PD assay, followed by immunoblotting using the indicated antibodies. **C** HEK293T cells were transfected with STING-Strep, TBK1-GST, and increasing amounts of DP71L-Flag plasmids. At the 24 hpt, cells were stimulated with 4 µg/mL of cGAMP for an additional 12 h. WCLs were then subjected for a Strep PD assay, followed by immunoblotting with the specified antibodies. **D** 3D4/21 cells were transfected with increasing amounts of DP71L-Flag plasmids. Subsequently, at the 24 hpt cells were stimulated with 4 µg/mL of cGAMP for an additional 12 h. WCLs were then utilized for the IP using STING antibody, followed by immunoblotting using the designated endogenous antibodies. **E** HEK293T cells, transfected with STING-Strep expression plasmids and increasing amounts of DP71L-Flag plasmids, were stimulated with 4 µg/mL of cGAMP. After 4 h, cell lysates were separated using SDD-AGE (top) or SDS-PAGE (bottom) and probed with the indicated antibodies. **F** PK-15 cells were transfected with control or different amounts of DP71L-Flag plasmids (100 and 200 ng). After 24 h, cells were stimulated with 4 µg/mL of cGAMP for an additional 12 h. Subsequently, the cells were fixed with 4% paraformaldehyde and prepared for confocal imaging. In immunoblot data, protein sizes are expressed in kDa. All the data are representative of at least three independent experiments, each with similar results.

To understand the DP71L interacting region of STING, we constructed expression plasmids encoding STING domains (Figure 4A), such as transmembrane (TM) domains, the dimerization domain (DD), the cyclic dinucleotide binding domain (CBD), and the C-terminal tail (CTT) containing the PXPLRXD conserved motif which interacts with TBK1 at the Golgi interface to induce IFN-I [37]. We conducted an immunoprecipitation experiment using GST-tagged STING domains (aa 1–145, aa 1–185, and aa 1–340) together with DP71L. Interestingly, we found that DP71L interacted specifically with the STING CTT (Figure 4B). Considering the direct interaction between the TBK1 and CTT of STING, which plays a pivotal role in activating the IFN-I signaling pathway, we performed a competition assay to thoroughly examine the potential inhibitory effect of DP71L on the binding of TBK1 to STING. Transfection with DP71L effectively inhibited the interaction between STING and TBK1 in HEK293T cells (Additional files 3A and B). In addition, this inhibitory effect was enhanced dose-dependently by increasing the concentration of DP71L (Figure 4C). Similar results were obtained in 3D4/21 cells (Figure 4D). Stable 3D4/21 cells infected with HSV-GFP (MOI = 1.0) were collected at 0, 6, 12, and 18 hpi. Immunoprecipitation with STING, followed by immunoblot analysis revealed a noticeable decrease in binding between endogenous TBK1 and STING (Additional file 3C). These results provide conclusive evidence that DP71L effectively inhibits the interaction between STING and TBK1. Stimulation with cGAMP induces STING polymerization independently of the STING CTT [38, 39]. To assess the effect of DP71L binding to CTT on STING polymerization, we performed a SDD-AGE assay, which revealed that a dose-dependent increase in DP71L had no impact on STING polymerization (Figure 4E and Additional file 3D). Confocal microscopy images clearly demonstrated formation of STING/TBK1 puncta. Interestingly, the presence of DP71L impaired formation of STING/TBK1 puncta, but had no effect on aggregated forms of STING (Figure 4F and Additional file 3E). These findings indicate that DP71L selectively targets the CTT of STING without disrupting STING activation. This interaction then inhibits the STING and TBK1 interaction.

### Mutations at P371, L374, and R375 of STING abolish its interaction with DP71L

**Figure 5:**
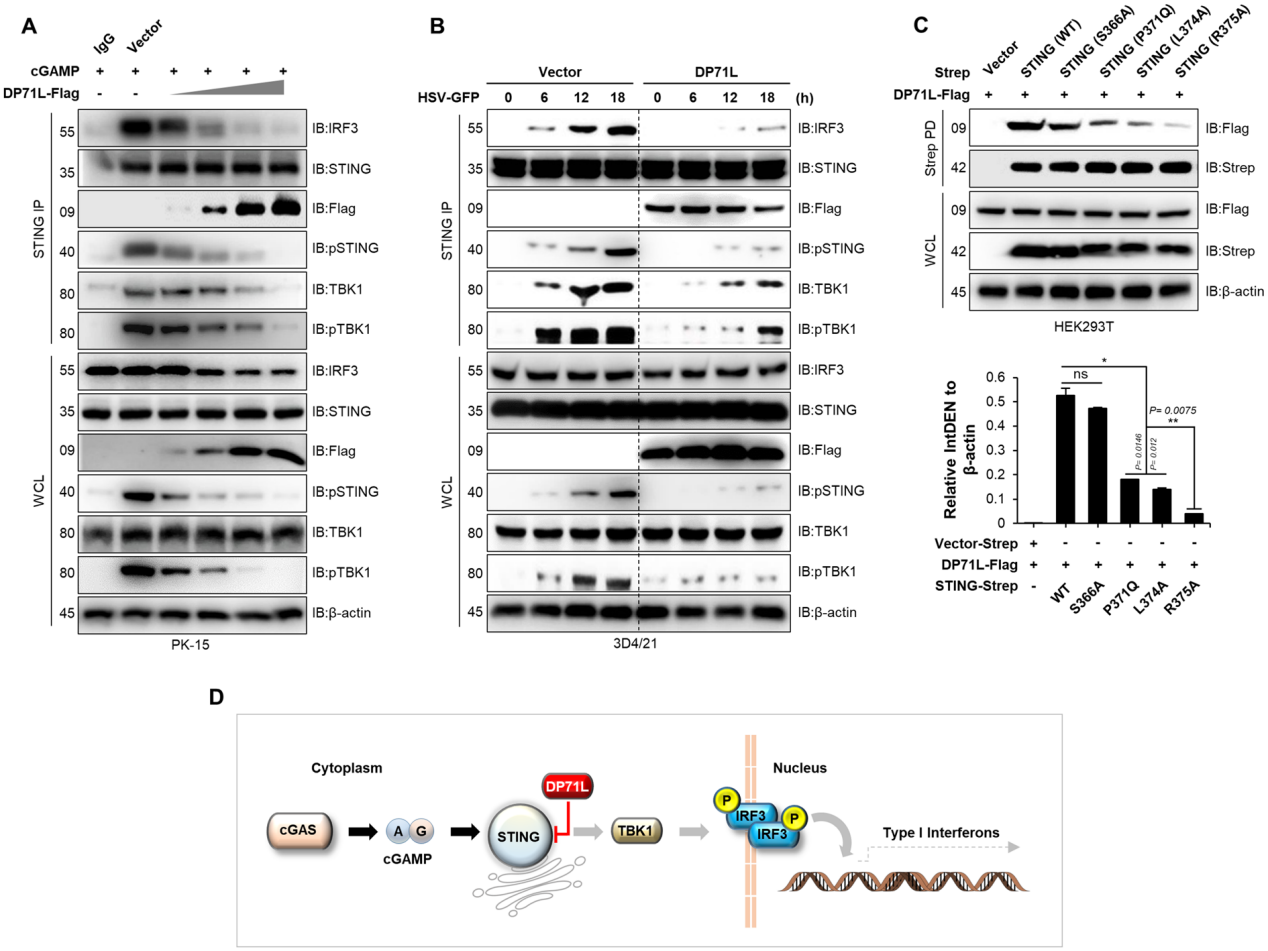
**The TBK1 binding motif of STING, not IRF3, determines the DP71L-STING interaction. A** PK-15 cells were transfected with DP71L-Flag plasmids in dose-dependent manner. After 24 h, cells were stimulated with 4 µg/mL of cGAMP for an additional 12 h. Subsequently, the WCLs were subjected to STING IP and bound proteins were detected by immunoblotting with indicated antibodies. **B** DP71L-Flag and control vector stably expressing 3D4/21 cells were infected with HSV-GFP (MOI = 1.0) and harvested at the indicated time points. WCLs were subjected to IP with endogenous STING and associated protein levels were detected by immunoblotting. **C** HEK293T cells were transfected with control, DP71L-Flag, and Strep-tagged STING mutant plasmids (S366A, P371Q, L374A, and R375A). Strep PD assay was performed, followed by immunoblotting with indicated antibodies. **D** Graphical summary of DP71L immune evasion. ASFV DP71L evades antiviral immune responses targeting the central immune molecule STING. Data are representative of at least two independent experiments, each with similar results. Protein sizes are expressed in kDa. The values are expressed as means ± SD for two biological replicates. Student’s *t*-test: *, *P* < 0.05; **, *P* < 0.01; ns, not significant.

The interaction between TBK1 and the STING CTT facilitates STING phosphorylation, specifically at S366 [39–41]. To investigate the potential inhibitory effect of DP71L on the interaction between phosphorylated STING (pSTING) and IRF3, we performed an endogenous immunoprecipitation assay using cGAMP-stimulated PK-15 cells. Increasing doses of DP71L reduced pSTING levels significantly, thereby diminishing the STING-IRF3 interaction (Figure 5A). This inhibitory effect was further confirmed in stable 3D4/21 cells infected with HSV-GFP (MOI = 1.0), as DP71L-expressing cells exhibited a time-dependent decrease in pSTING levels compared with control cells, resulting in a reduced endogenous STING-IRF3 interaction (Figure 5B). However, it is still not clear whether binding of DP71L to the CTT of STING inhibits its interaction with IRF3. To explore this further, we performed a mutation analysis of STING to accurately identify the specific binding site between STING and DP71L. Immunoprecipitation assays revealed that DP71L interacts with STING through the TBK1 binding site, which includes residues P371, L374, and R375 [39]. Notably, phosphorylation site S366, which is responsible for CTT phosphorylation and IRF3 binding, did not contribute to the interaction between DP71L and STING (Figure 5C). These findings strongly suggest that binding of DP71L to the STING CTT specifically inhibits the interaction between STING and TBK1, leading to suppression of TBK1 autophosphorylation, STING phosphorylation, and subsequent binding between pSTING and IRF3 (Figure 5D).

### DP71L knockdown promotes antiviral gene levels in ASFV-infected cells

**Figure 6:**
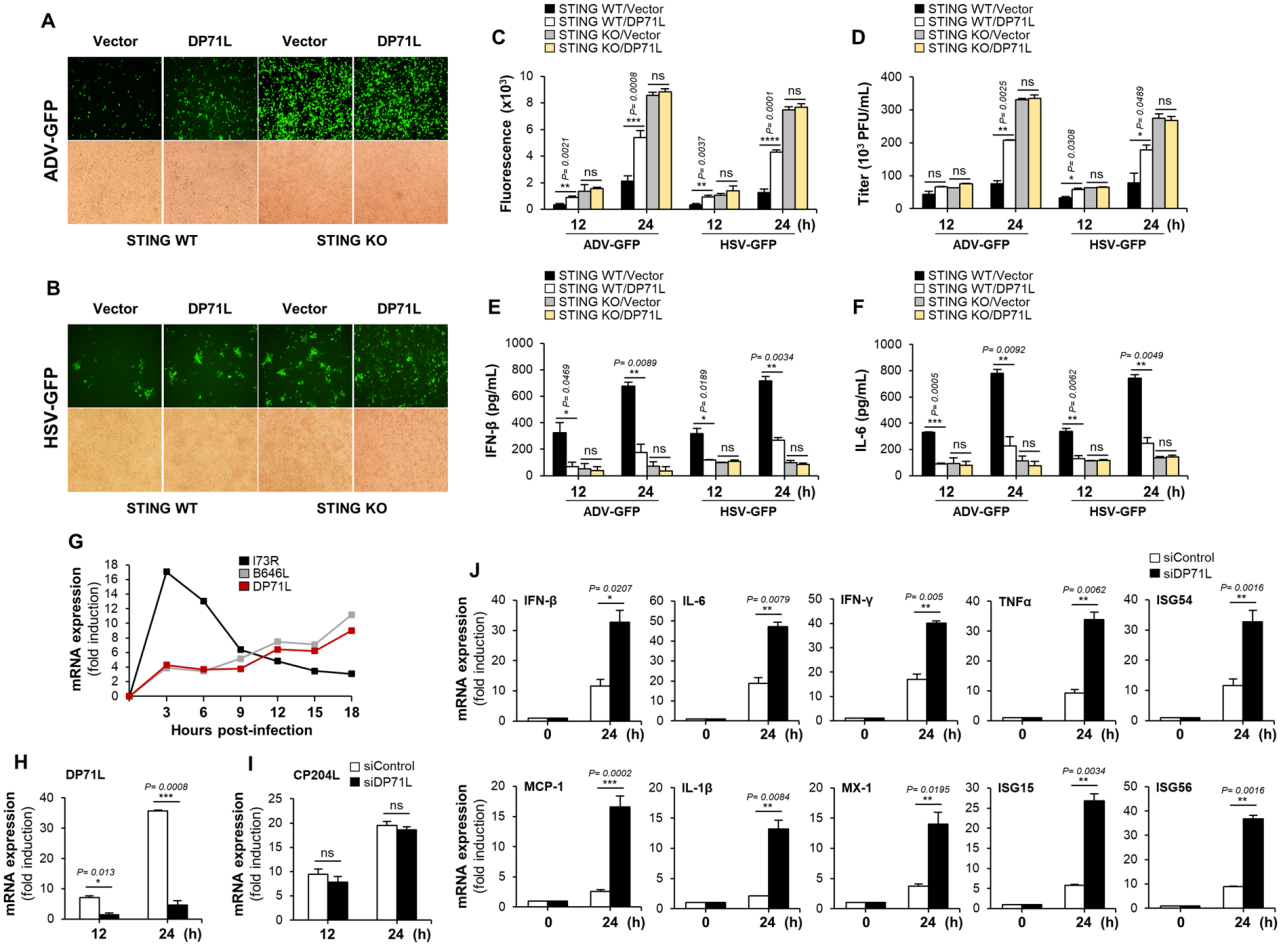
**STING is essential for the DP71L antiviral effect, and its knockdown enhances antiviral gene expression.** **A**, **B** STING KO and STING WT HeLa cells were transfected with DP71L-Flag or control vector and after 12 h, cells were infected with ADV-GFP **A** or HSV-GFP **B** at a 1 MOI. Fluorescence images were captured at 24 hpi using fluorescence microscopy. **C** Fluorescence levels were measured at both 12 and 24 hpi with a fluorescence modulator. **D** Viral titers were determined by standard plaque assays in A549 and Vero cells using the virus-infected cell supernatants harvest at indicated time points. **E**, **F** Porcine IFN-β and IL-6 concentrations in the cell culture supernatant collected at 12 and 24 hpi were quantified using ELISA. **G** Primary PAM cells were infected with ASFV an MOI of 0.5. Cell pellets were harvested at 0, 3, 6, 9, 12, 15, and 18 hpi, and RNA was extracted. DP71L, I73R and B646L gene transcriptions were determined by qRT-PCR. **H**, **I** Primary PAM cells were transfected with siDP71L or siControl for 6 h and then cells were infected with ASFV at an MOI of 0.5. Transcription levels of the indicated genes were examined at the 12 and 24 hpi. **J** Primary PAM cells were maintained in 24-well cell culture plate and cells were transfected with siDP71L or siControl for 6 h. Subsequently, cells were infected with ASFV (MOI = 0.5). Transcription levels of the indicated antiviral genes were examined at indicated time points. The data represent at least two independent experiments with similar results, and the values are expressed as means ± SD for two biological replicates. Student’s *t*-test, *, *P* < 0.05; **, *P* < 0.01; ***, *P* < 0.001; ns, not significant.

To validate the antagonistic effect of DP71L on antiviral immunity, we assessed replication of GFP-tagged DNA viruses, as well as cytokine levels in STING KO HeLa cells expressing DP71L. DP71L expression plasmids were transfected into STING KO and STING WT HeLa cells, which were then infected with ADV-GFP and HSV-GFP. GFP fluorescence and virus titers were then measured. The results showed that STING KO cells exhibited higher GFP absorbance and virus titers than STING WT cells (Figures 6A–D). Importantly, there was no significant difference in fluorescence and virus titers between the control and DP71L-containing STING KO cells. Moreover, secretion of IFN-β and IL-6 into the supernatants of virus-infected STING KO cells was lower than in those from STING WT cells, with no significant difference between the control and DP71L-transfected STING KO cells (Figures 6E and F). This indicates that STING is essential for the antiviral effects of DP71L. Next we investigated the transcription kinetics of DP71L by isolating total RNA from primary PAM infected with ASFV (Korea/wild boar/Hwacheon/2020–2287 strain). The results indicate that the transcription pattern of DP71L closely resembles that of B646L, a late-transcribed gene, but differs from that of I73R, an early-transcribed gene (Figure 6G). This observation confirms that DP71L is a late-transcribed gene of ASFV [33]. To investigate the immunomodulatory functions of DP71L during ASFV infection, we utilized DP71L-specific siRNA, siDP71L to knock-down DP71L in primary PAM upon infecting them with ASFV. The efficiency of DP71L knockdown was nearly 87% at 24 hpi (Figure 6H) and it did not affect the expression of ASFV CP204L gene (Figure 6I), suggesting that the siRNA treatment has no effect on replication of the infected ASFV. Additionally, qRT-PCR analysis of siRNA-treated ASFV-infected primary PAM cells revealed higher expression of antiviral genes in siDP71L cells than in control cells (siControl) (Figure 6J).

## Discussion

ASFV employs a range of sophisticated mechanisms to modulate the host immune system, particularly targeting the cGAS-STING signaling pathway to suppress IFN-1 production [42]. ASFV proteins such as QP383R inhibit cGAS enzymatic activity, reducing cGAMP production [43], while EP364R and C129R degrade cGAMP [23], further impairing the pathway. Additionally, B175L competes with cGAMP for binding to STING, disrupting the cGAMP-STING interaction [32]. Several ASFV gene products target TBK1 promoting its lysosomal degradation [24, 44], or inhibiting phosphorylation [45]. Additionally, MGF505-7R and E301R inhibit IRF3 nuclear translocation, further suppressing its role in antiviral signaling [46, 47]. Furthermore, ASFV genes employ multiple strategies to suppress STING function, including inhibiting its translocation from the endoplasmic reticulum to the Golgi apparatus [48, 49], promoting its degradation via autophagy or proteasomal pathways [21, 29, 31, 50], and impairing its ubiquitination, dimerization, and oligomerization, thereby reduces the formation of STING-TBK1-IRF3 complex [51]. However, the involvement of ASFV genes in targeting STING-CTT to suppress innate immune responses remains unexplored.

In this study, we describe a new molecular mechanism involving ASFV DP71L that may increase ASFV virulence by facilitating evasion of host interferon signaling. Previous research shows that ASFV DP71L interacts with PP1 to dephosphorylate eIF2α, leading to restoration of host protein translation and apoptosis [33]. In addition, deletion of DP71L has been studied as a promising target for development of a safe and efficacious ASFV vaccine. One such study reports that pigs inoculated with short form of DP71L (NL-S) gene-deleted recombinant ASF viruses (E70/43) show reduced virulence and are protected against challenge with the virulent parental European isolate E70 [52]. By contrast, another study shows that inoculation of pigs with DP71L gene-deleted ASFV Malawi Lil-20/1 (Mal) and Pretoriuskop/96/4 (Pr4) strains did not attenuate virulence, resulting in 100% mortality [53]. A recent attempt to develop a recombinant live attenuated vaccine against ASFV showed that deletion of DP71L, along with DP148R and DP96R, from the highly virulent isolate ASFV CN/GS/2018 (ASFV-GS) significantly attenuated virulence in pigs [34]. These reports suggest that DP71L is involved in ASFV virulence, and may be a target for vaccine development. However, the exact molecular mechanism remained unknown.

Upon recognizing viral double-stranded DNA, cGAS synthesizes cGAMP, which binds to STING on the ER membrane. STING’s N-terminal region contains four transmembrane segments that anchor it to the ER, while the cytoplasmic CTT plays a pivotal role in mediating downstream signaling [54]. Upon activation by DNA or cGAMP, STING undergoes polymerization and utilizes its CTT to recruit and activate TBK1 and IRF3. This activation initiates a signaling cascade that culminates in the production of IFN-I, driving the antiviral response [41, 55]. A previous report demonstrates that mutations in the residues at the interface between STING (P371Q, L374A, and R375A) and TBK1 (Y577A, N578A, and Q581A) disrupts cGAMP-induced expression of IFN-β, which is associated with activation of TBK1, STING and IRF3 [39]. These findings highlight the crucial role of these residues in the signaling functions of STING and TBK1. Additionally, the interaction between TBK1 and the CTT domain of STING facilitates STING phosphorylation, particularly at S366, allowing subsequent recruitment and phosphorylation of IRF3 by TBK1. The CTT domain of phosphorylated STING serves as a platform for the concurrent interaction between TBK1 and IRF3, thereby facilitating delivery of IRF3 to TBK1 for phosphorylation [39].

Here, we showed that the conserved PP1 motif of DP71L interacts specifically with the CTT of STING, along with the surprising observation of an interaction between DP71L and STING at the TBK1 binding site, encompassing residues P371, L374, and R375. Notably, increasing amounts of DP71L competitively inhibited the transient and endogenous interaction between STING and TBK1 (Figures 4A–D). Interestingly, SDD-AGE and confocal microscopy assays provide compelling evidence that DP71L does not affect STING polymerization (Figures 4E and F). These findings strongly suggest that DP71L selectively targets STING without interfering with its polymerization. As expected, this interaction results in a decrease in TBK1 autophosphorylation, STING phosphorylation, and decrease in the endogenous interaction between pSTING and IRF3 (Figures 5A and B). By contrast, phosphorylation site S366, which is involved in STING phosphorylation and IRF3 binding, did not contribute to the DP71L-STING interaction (Figure 5C). Next, we found that STING is the actual target of ASFV DP71L for immune evasion using STING KO cells and finally, the knockdown of DP71L using siRNA significantly upregulated antiviral gene expression in ASFV-infected cells (Figure 6).

By the time late viral genes are expressed, ASFV has firmly established itself within the host cell and initiated replication. At this stage, the virus prioritizes ensuring its survival and facilitating its spread by evading the host's immune defenses. Late gene products play a critical role in this process, often functioning as immune evasion tools that specifically target and suppress early innate immune responses, which would otherwise impede viral replication. Our findings demonstrate that DP71L, a late-transcribed gene of ASFV, possesses the ability to inhibit the host's early immune responses. Given that multiple late-transcribed ASFV genes target the cGAS-STING pathway to suppress early IFN responses [32, 43, 49], we propose that ASFV employs a multifaceted strategy to evade host immune defenses, enabling successful viral replication and dissemination.

In summary, the effect of DP71L on the virulence of ASFV has been showed through in vivo pig experiments. However, the exact molecular mechanism of DP71L remains unknown. In this study, we found a novel molecular mechanism for DP71L acts as an IFN-I antagonist. DP71L interacts with the CTT region of STING, thereby disrupting STING-mediated downstream transmission of antiviral signals involving TBK1 and IFR3. These findings comprehensively expand our understanding of diverse mechanisms underlying ASFV pathogenesis and provide new avenues for studying viral attenuation, as well as for development of ASFV vaccines.

## Supplementary Information

**Additional file 1. DP71L downregulates antiviral immune responses in PK-15 cells. A** DP71L-Flag expression in transfected-PK-15 cells. **B, D, F** PK-15 cells were transfected with DP71L-Flag or control vector plasmids for 12 h and cells were infected with ADV-GFP, HSV-GFP, or VACV-GFP. The fluorescence images of virus replications were taken at 24 hpi using fluorescence microscopy and quantified at 12 and 24 hpi using the fluorescence modulator. Virus titers were measured by standard plaque assay in A549 and Vero cells. **C, E, G** Porcine IFN-β and IL-6 concentrations in the cell culture supernatants that were collected at 12 hpi and 24 hpi were analyzed by respective ELISA. The data presented are representative of at least two independent experiments, each showing similar results. Protein sizes are expressed in kDa. The values provided represent the means ± SD of two biological replicates. Student’s *t*-test: *, *P* < 0.05; **, *P* < 0.01; ***, *P* < 0.001; ns, not significant

**Additional file 2. DP71L inhibits antiviral immune responses in MA104 cells. A–C **DP71L-Flag expression in stable MA104 cells, stable 3D4/21 cells, and stable PIB cells. **D, F, H** MA104 cells stably expressing DP71L-Flag or control vector were infected with ADV-GFP, HSV-GFP, or VACV-GFP. The GFP images were captured at 24 hpi using fluorescence microscopy and quantified at 12 and 24 hpi using the fluorescence modulator. Virus titers of each sample were determined by standard plaque assay in A549 and Vero cells. **E, G, I** IFN-β and IL-6 concentrations in the cell culture supernatants that were collected at 12 hpi and 24 hpi were estimated by ELISA. The data presented are representative of at least two independent experiments, each showing similar results. Protein sizes are expressed in kDa. The values provided represent the means ± SD of two biological replicates. Student’s *t*-test: *, *P* < 0.05; **, *P* < 0.01; ***, *P* < 0.001.

**Additional file 3. DP71L disrupts the interaction between STING and TBK1 but does not affect STING polymerization. A **HEK293T cells were transfected with STING-Strep, TBK1-GST, and DP71L-Flag plasmids. After 24 hpt, the cells were stimulated with 4 µg/mL of cGAMP for an additional 12 h. WCLs were then used for a Strep PD assay, followed by immunoblotting with the specified antibodies. **B** HEK293T cells were transfected with STING-Strep, DP71L-Flag, and respective control plasmids. WCLs were then subjected to Strep PD, followed by immunoblotting using the indicated antibodies. **C** DP71L-Flag or control vector stably expressing 3D4/21 cells were infected with HSV-GFPand collected at the designated time points. WCLs were immunoprecipitated using STING antibody and analyzed by immunoblotting. **D **Integrated density (IntDen) analysis of Figure 4E. **E** Graph illustrating the STING-TBK1 puncta fluorescence intensities in Figure 4F. The data presented are representative of at least two independent experiments, each showing similar results. Protein sizes are expressed in kDa. The values provided represent the means ± SD of two biological replicates. Student’s t-test: ns, not significant.

## Data Availability

The data that support the findings of this study are available within the main body of the manuscript and additional files. Further inquiries can be directed to the corresponding author.
