# Supplementary figures and images for: Inhibition of STING-mediated type I IFN signaling by African swine fever virus DP71L
Source: Vet Res. 2025 Feb 4;56:27. doi: 10.1186/s13567-025-01474-3 (PMC11796124; doi:10.1186/s13567-025-01474-3)

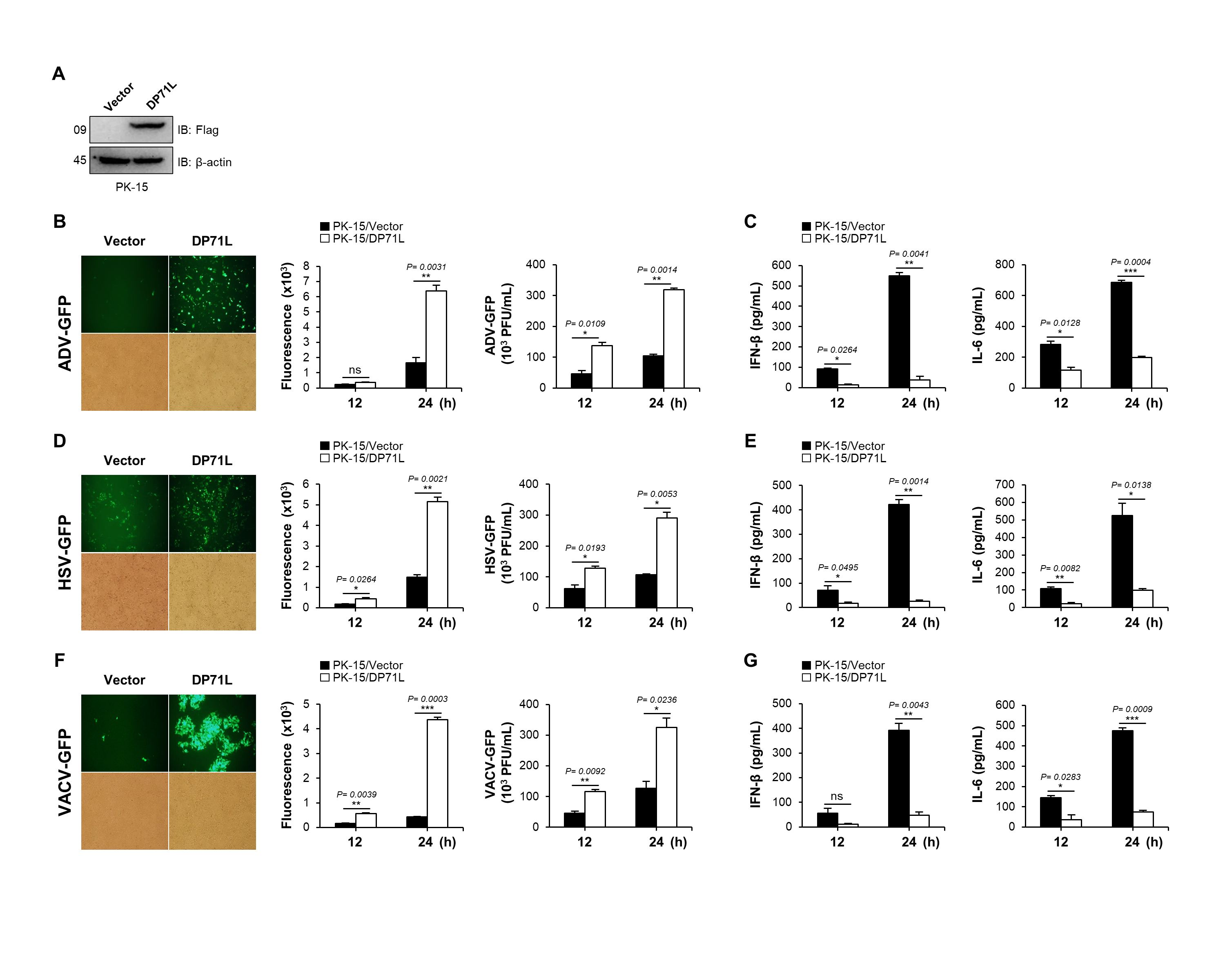

Supplement: Supplementary file 1 — Additional file 1. DP71L downregulates antiviral immune responses in PK-15 cells. A DP71L-Flag expression in transfected-PK-15 cells. B, D, F PK-15 cells were transfected with DP71L-Flag or control vector plasmids for 12 h and cells were infected with ADV-GFP, HSV-GFP, or VACV-GFP. The fluorescence images of virus replications were taken at 24 hpi using fluorescence microscopy and quantified at 12 and 24 hpi using the fluorescence modulator. Virus titers were measured by standard plaque assay in A549 and Vero cells. C, E, G Porcine IFN-β and IL-6 concentrations in the cell culture supernatants that were collected at 12 hpi and 24 hpi were analyzed by respective ELISA. The data presented are representative of at least two independent experiments, each showing similar results. Protein sizes are expressed in kDa. The values provided represent the means ± SD of two biological replicates. Student’s t-test: *, P < 0.05; **, P < 0.01; ***, P < 0.001; ns, not significant [file 13567_2025_1474_MOESM1_ESM.tif]

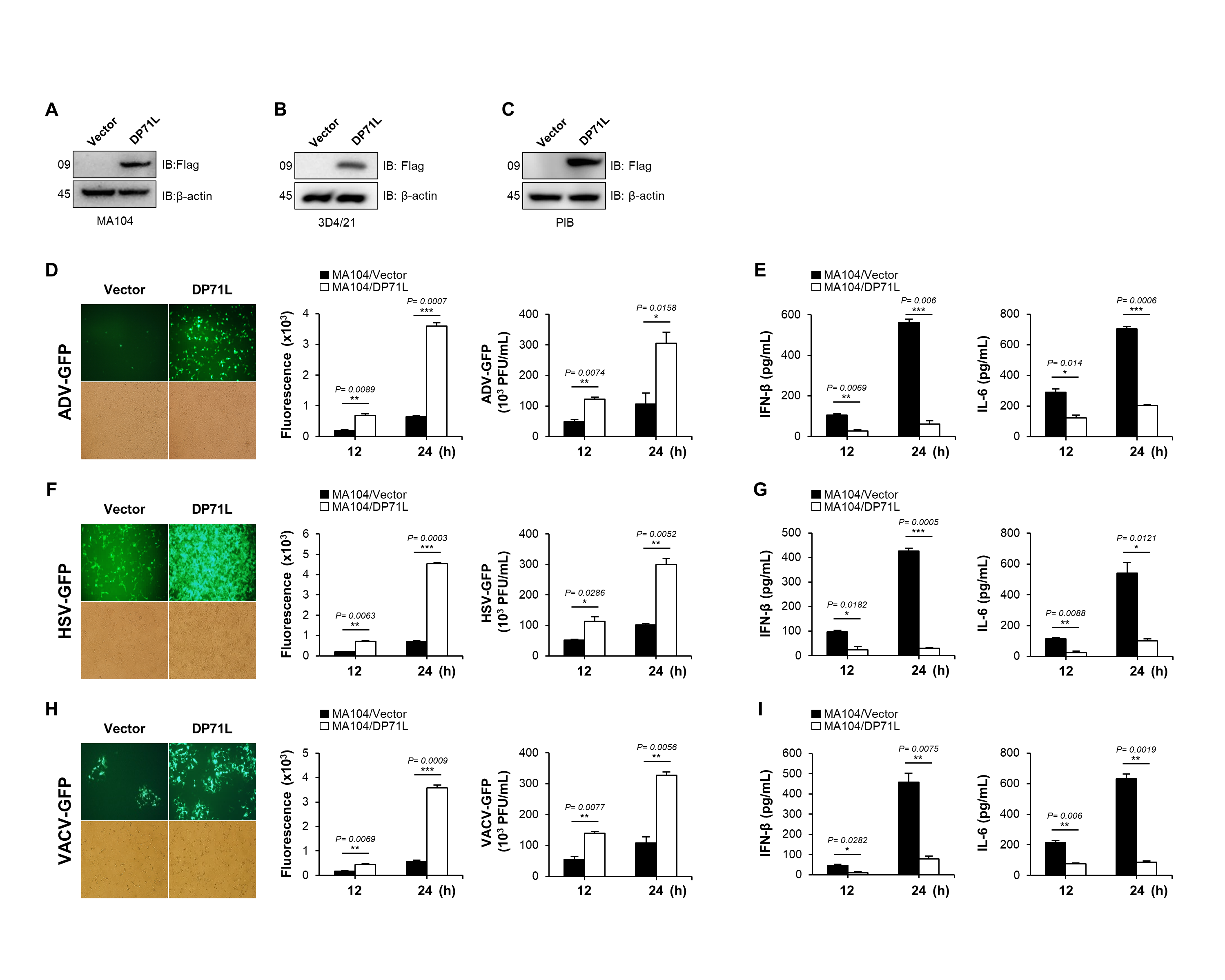

Supplement: Supplementary file 2 — Additional file 2. DP71L inhibits antiviral immune responses in MA104 cells. A–C DP71L-Flag expression in stable MA104 cells, stable 3D4/21 cells, and stable PIB cells. D, F, H MA104 cells stably expressing DP71L-Flag or control vector were infected with ADV-GFP, HSV-GFP, or VACV-GFP. The GFP images were captured at 24 hpi using fluorescence microscopy and quantified at 12 and 24 hpi using the fluorescence modulator. Virus titers of each sample were determined by standard plaque assay in A549 and Vero cells. E, G, I IFN-β and IL-6 concentrations in the cell culture supernatants that were collected at 12 hpi and 24 hpi were estimated by ELISA. The data presented are representative of at least two independent experiments, each showing similar results. Protein sizes are expressed in kDa. The values provided represent the means ± SD of two biological replicates. Student’s t-test: *, P < 0.05; **, P < 0.01; ***, P < 0.001. [file 13567_2025_1474_MOESM2_ESM.tif]

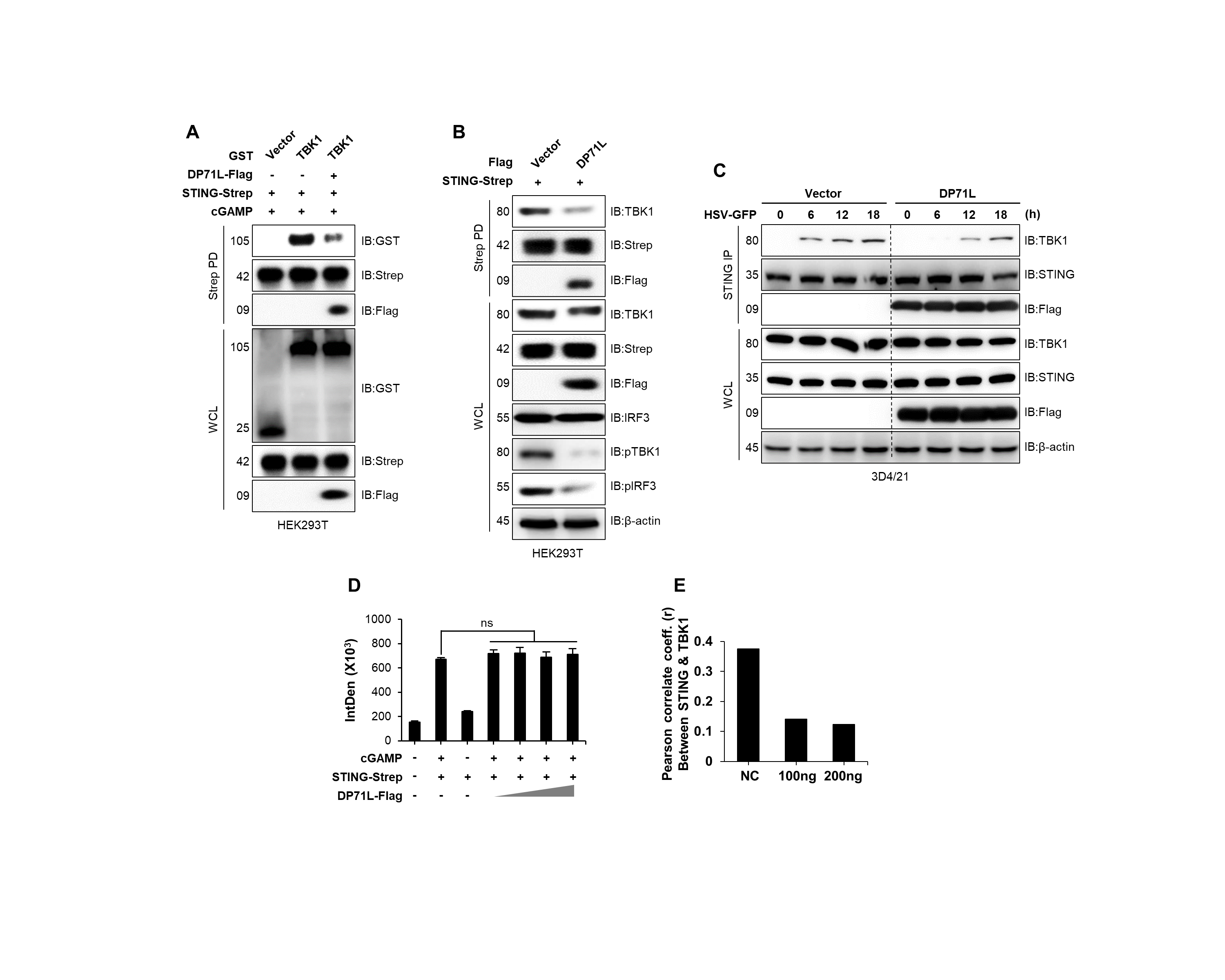

Supplement: Supplementary file 3 — Additional file 3. DP71L disrupts the interaction between STING and TBK1 but does not affect STING polymerization. A HEK293T cells were transfected with STING-Strep, TBK1-GST, and DP71L-Flag plasmids. After 24 hpt, the cells were stimulated with 4 µg/mL of cGAMP for an additional 12 h. WCLs were then used for a Strep PD assay, followed by immunoblotting with the specified antibodies. B HEK293T cells were transfected with STING-Strep, DP71L-Flag, and respective control plasmids. WCLs were then subjected to Strep PD, followed by immunoblotting using the indicated antibodies. C DP71L-Flag or control vector stably expressing 3D4/21 cells were infected with HSV-GFPand collected at the designated time points. WCLs were immunoprecipitated using STING antibody and analyzed by immunoblotting. D Integrated density (IntDen) analysis of Figure 4E. E Graph illustrating the STING-TBK1 puncta fluorescence intensities in Figure 4F. The data presented are representative of at least two independent experiments, each showing similar results. Protein sizes are expressed in kDa. The values provided represent the means ± SD of two biological replicates. Student’s t-test: ns, not significant. [file 13567_2025_1474_MOESM3_ESM.tif]
